# Adaptive Segmented Aggregation and Rate Assignment Techniques for Flexible-Length Polar Codes

**DOI:** 10.3390/e26070584

**Published:** 2024-07-09

**Authors:** Souradip Saha, Shubham Mahajan, Marc Adrat, Wolfgang Gerstacker

**Affiliations:** 1 Fraunhofer Institute for Communication, Information Processing and Ergonomics, Fraunhoferstraße 20, 53343 Wachtberg, Germany; 2 Institute for Digital Communications, Friedrich-Alexander-Universität, Erlangen-Nürnberg, Cauerstr. 7, 91058 Erlangen, Germany; shubham.mahajan@fau.de (S.M.); wolfgang.gerstacker@fau.de (W.G.)

**Keywords:** polar codes, puncturing, shortening, adaptive segmented aggregation, rate-assignment

## Abstract

Polar codes have garnered a lot of attention from the scientific community, owing to their low-complexity implementation and provable capacity achieving capability. They have been standardized to be used for encoding information on the control channels in 5G wireless networks due to their robustness for short codeword lengths. The conventional approach to generate polar codes is to recursively use 2×2 kernels and polarize channel capacities. This approach however, has a limitation of only having the ability to generate codewords of length $N_{orig}=2^{n}$ form. In order to mitigate this limitation, multiple techniques have been developed, e.g., polarization kernels of larger sizes, multi-kernel polar codes, and downsizing techniques like puncturing or shortening. However, the availability of so many design options and parameters, in turn makes the choice of design parameters quite challenging. In this paper, the authors propose a novel polar code construction technique called Adaptive Segmented Aggregation which generates polar codewords of any arbitrary codeword length. This approach involves dividing the entire codeword into smaller segments that can be independently encoded and decoded, thereby aggregated for channel processing. Additionally a rate assignment methodology has been derived for the proposed technique, that is tuned to the design requirement.

## 1. Introduction

Polar codes, a set of linear block error correcting codes, first introduced in [1], offer many advantages for channel coding of information bits to be transmitted across a noisy channel, such as low complexity, provable capacity achieving capability, and robust characteristics for short codeword lengths. Therefore, they are standardized to be used for 5G wireless control channels [2]. The recursive structure of channel polarization is further used to design the successive cancellation (SC) decoding technique [1] with multiple variations like SC List (SCL) decoding, SCL+CRC decoding [3], and SC Flip (SCF) decoding [4], etc. Additionally a soft-input soft-output (SISO)-based belief propagation (BP) decoding of polar codes is also well established [5].

Since polar codes have been conventionally based on polarization of sets of 2 bits using a 2×2 polarization kernel, there exists a limitation: the ability to generate only codewords of length $N_{orig}=2^{n}$ form ($N_{orig}$ is used only for polar codewords of length $2^{n}$ form in this paper). However, since the concept of channel polarization is not limited to any specific kernel size, one may extend it to 3×3, 4×4, 5×5 kernels and so on, thus laying the foundation for generating codewords of $3^{n}$ [6,7,8,9] or effectively, any generic $a^{n}$ for any arbitrary a∈N. Thereafter, multiple polarization kernels can be combined within one polarization circuit, thus creating multi-kernel polar codes (MKPCs) [7,10,11,12,13,14,15]. These polar code construction techniques provide the ability to design non-conventional polar codes with a wide range of possible codeword lengths. In addition to multiple possibilities of polarization kernels, there exist multiple downsizing techniques, namely, puncturing [16,17] and shortening [18], that are well established in the literature. Prior research has been conducted by our research team to combine the different aforementioned techniques for improving the design and generation of polar codes [19,20,21].

In this paper, we provide a novel approach to generating polar codes, where the required codeword is divided such that each segment can be encoded and decoded independently of each other, and then, concatenated together. Since the concatenation is a linear approach, the effective codeword length can be any arbitrary integer value, thus resulting in a codeword length that is in no way limited by the underlying polarization kernels or downsizing techniques. This is a major design improvement over the well-known polar code construction techniques.

The rest of the paper is organized as follows. In Section 2, some preliminary information is provided. Section 3 introduces the concept of Adaptive Segmented Aggregation (ASA) and provides a detailed design methodology along with corresponding segmentation rules. Encoding and decoding using the ASA method are detailed. Additionally, two rate assignment techniques (RATs) are proposed, which are dependent on the desirable coderate. In Section 4, assessment of the proposed ASA technique is analyzed, first with respect to the impact on design complexity compared to downsizing techniques, and thereafter, with respect to the bit error-rate (BER) performance curves for validation as well as the impact of various coderates and corresponding RATs. Section 5 and Section 6 summarize this paper with some ideas on future research work and conclusions, respectively.

## 2. Preliminaries

### 2.1. Channel Polarization—2×2 Kernel

The conventional approach of channel polarization, as proposed in [1], utilizes a 2×2 kernel to polarize 2-bit channels of equal capacities to 2-virtual-bit channels of unequal capacities. Such a 2×2 kernel is then recursively used (interconnected) to generate a polarization circuit, in order to design $2^{n}$-bit channels, by using *n* stages of channel polarization, with unequal polarization. The reader is recommended to refer to [1] for details on the concept of channel polarization.

### 2.2. Polar Encoding and Decoding

In this section, we provide a brief overview of polar encoding and decoding, used within the scope of this paper.

#### 2.2.1. Encoding

Polar encoding is performed using the polarization developed on the basis of unequal channel polarization. Since all virtual bit channels have unequal capacity (owing to channel capacity), the idea is to split the bit channels into two categories—one with frozen bits and one with information bits. The frozen bits correspond to a known vector (typically a zero-vector), such that the desired coderate $R_{d}$ for codeword length *N* is maintained, i.e.,

(1) $$\left\lceil \mathrm{frozen}\;\mathrm{bits}\right\rceil =\left(1-R_{d}\right)\cdot N_{orig}$$

and

(2) $$\left\lfloor \mathrm{information}\;\mathrm{bits}\right\rfloor =R_{d}\cdot N_{orig}$$

The frozen bits are assigned to bit indices which have the highest so-called *Z*-values (lowest channel capacities) after channel polarization, whereas the information bits are assigned to bit indices which have the lowest *Z*-values (highest channel capacities) after channel polarization. Thereafter, the vectors of frozen and information bits are concatenated to an input bit vector $\vec{U}$ and transformed using the generator matrix *G* to obtain the output bit vector $\vec{X}$, as follows:

(3) $$\vec{X}=G\cdot \vec{U}$$

For additional details regarding the *Z*-values (Bhattacharya parameter), generator matrix, bit selection, and polar encoding, the reader is recommenced to refer to [1].

#### 2.2.2. Successive Cancellation Decoding

The recursive structure of channel polarization can be exploited to develop an SC decoder, where the bits are decoded one after the other and the bit estimate is thereby used to decode consecutive bits. Within the scope of this paper, polar decoding is implemented only using an SC decoder. For further details on the decoding mechanism, the reader is recommended to refer to [1].

**Figure 1:**
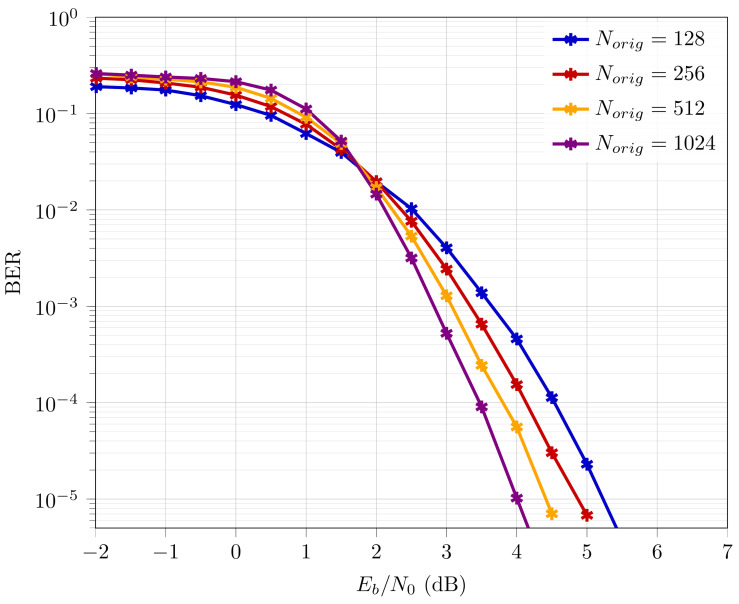
BER performance of polar codes for $N_{orig}=2^{n}$ at $R_{d}=0.5$.

Figure 1 provides the comparative BER performance for polar codes of different codeword lengths at coderate $R_{d}=0.5$. Here, the typical behavior of SC polar decoding is observed, where the error-rate performance for polar codes with higher codeword lengths eventually outperforms their counterparts with lower codeword lengths.

### 2.3. Downsizing Techniques

In this section, we provide a brief overview of the downsizing techniques, namely, puncturing and shortening, for polar encoding and decoding, used within the scope of this paper.

#### 2.3.1. Puncturing

Puncturing is a well-known downsizing technique, where the punctured codeword bits are not transmitted across the channel from the transmitter to the receiver. The decoder at the receiver typically handles such punctured bits as bits erased over the channel. From the polarization point of view, to minimize loss of capacity by puncturing, it is beneficial to use the virtual-bit channels with minimum channel capacities (i.e., bits with maximum *Z*-values) for puncturing. Puncturing as a downsizing technique for polar codes is well-known in the literature [16,17]. The puncturing scheme used here is the same as that used in [19], and the reader is recommended to refer to it for further details.

#### 2.3.2. Shortening

Shortening is another well-known downsizing technique, where shortened codeword bits are not transmitted across the channel from the transmitter to the receiver. However, unlike the puncturing scheme, the decoder at the receiver typically handles shortened bits as a priori bits or bits already known with full confidence. From the polarization point of view, to minimize loss of capacity by shortening, it is beneficial to use the virtual bit channels with maximum channel capacities (i.e., bits with minimum *Z*-values) for shortening. Like puncturing, shortening as a downsizing technique for polar codes is also well-known in the literature [18]. The shortening scheme used here is the same as that used in [19], and the reader is recommended to refer to it for further details.

## 3. Novel Adaptive Segmented Aggregation of Polar Codes

This section introduces the novel polar code construction technique Adaptive Segmented Aggregation (ASA), using which, polar codes of variable codeword lengths are designed. This approach allows one to segment a given codeword length into smaller segments that can be independently processed similar to polar codes of form $N_{orig}=2^{n}$ or $3^{n}$. The following subsections will describe the encoding/decoding process at transmitter/receiver for ASA-based polar codes.

### 3.1. ASA-Based Polar Encoding

Here, we outline the principles for dividing a desired codeword length into smaller codeword lengths, referred to as segments in this document, which are processed individually before being combined for transmission through the channel. To limit the scope of analysis, only kernels of size 2 are considered for the rest of this paper. Hence, encoder segments of form $N_{orig}=2^{n}$ are developed. However, the concept can easily be extended to any higher-order kernels as well.

**Figure 2:**
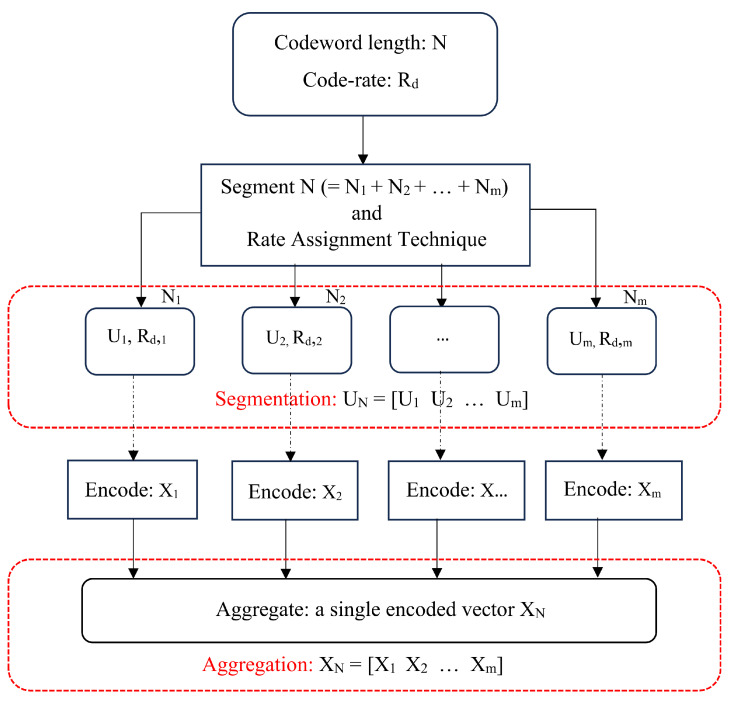
Block diagram for ASA encoding.

Figure 2 depicts a block diagram showing the process of encoding in the ASA approach for polar codes. It has two main blocks, namely, segmentation and aggregation. Given the system parameters, the segmentation block breaks the provided codeword length *N* into *m* segments such that the sum of the codeword lengths of *m* segments is *N*. This can be visualized as a super-generator matrix used to encode a concatenated input vector ${\vec{U}}_{N}$ to output vector ${\vec{X}}_{N}$, as follows:

(4) $${\vec{X}}_{N}=G_{N}\cdot {\vec{U}}_{N}$$

where

(5) $$G_{N}=\left(\begin{array}{c} G_{N_{1}\times N_{1}}\;0_{N_{1}\times N_{2}}\;\cdots \;0_{N_{1}\times N_{m}}\\ 0_{N_{2}\times N_{1}}\;G_{N_{2}\times N_{2}}\;\cdots \;0_{N_{2}\times N_{m}}\\ \vdots \;\vdots \;\vdots \;\vdots \\ 0_{N_{m}\times N_{1}}\;0_{N_{m}\times N_{2}}\;\cdots \;G_{N_{m}\times N_{m}} \end{array}\right)$$

is equivalent to the direct sum of square matrices representation over Galois Field 2 (Equation (10) of [22]), and

(6) $$N=\sum_{i=1}^{m}N_{i}$$

Thereafter, coderates $R_{d,m}$ for *m* segments are allocated as per corresponding rate assignment techniques (RATs). Upon segmentation and rate assignment, the segments are encoded similar to conventional polar codes of form $N_{orig}=2^{n}$. Finally, the encoded outputs $X_{m}$ of *m* segments are aggregated together to build a single encoded vector $X_{N}$. This vector is then transmitted via a given channel.

### 3.2. Segmentation Rules

In order to formalize the polar code construction technique using ASA, we develop some segmentation rules, detailed as follows:

1. The length of each segment should be an integer of power of the underlying kernel size. Since we limit our analysis to kernel size 2 only, each segment length should be of $2^{n}$ form.
  - Limiting case: n=0 is considered valid. This would result in a segment of one uncoded bit. This would aid in designing codewords of odd-integer length and it is uncoded since it will not be polarized.
2. Given an overall codeword length *N*, the length of the first segment $N_{1}$ is of form $2^{n}$ and its value is closest to and ≤N.
3. Similarly, the length of the second segment $N_{2}$ is of form $2^{n}$ and its value is closest to and $\leq (N-N_{1})$, i.e., it is desirable to have a larger codeword length for better error-rate performance (reasoning corresponds to observation from Figure 1).
4. Step 2 is repeated until the sum of the length of all segments ($N_{1}$, $N_{2}$, …, $N_{m}$) equals the overall codeword length *N*, i.e., $N=N_{1}+N_{2}+...+N_{m}$.

To understand the segmentation rules, let us consider an example: say the required codeword length N=20, the possibilities of segments are:

- N=16+4 (as per rule);
- N=8+8+4;
- N=4+4+4+4+4;
- N=16+2+2.

Polar codes provably achieve capacity for infinite codeword length, when the rate of polarization is maximum. Polar codes with longer codeword lengths exhibit better BER performance than their shorter counterparts, as observed in Figure 1. Extending this observation, it would be fair to assume that N=16+4 is expected to result in better BER performance compared to other choices. This is because the presence of the largest codeword length ($N_{1}=16$) improves the overall BER performance. This is validated in Section 4.2.1.

The segmentation rules have been developed in order to minimize the overall BER performance. Therefore, the segmentation rules emphasize the use of longer codeword lengths, to maximize the advantage of error correcting capabilities of polar codes with longer codeword lengths. To optimize the ASA design for frame error rate (FER), the segmentation rules need to be redefined. Since the combined set of segments are one frame, it would be instrumental to avoid very short segment codeword lengths, which would be the limiting factor for the overall FER performance. This is particularly critical with respect to the limiting case of segmentation rule 1, where the uncoded bit would be detrimental to the FER performance. The FER specific analysis is beyond the scope of this paper.

The following subsections describe the RATs to observe and evaluate performance variation by allocating the same or different coderates $R_{d,m}$ to the *m* segments, given the system’s overall coderate $R_{d}$.

### 3.3. Rate Assignment Techniques

The system parameter coderates significantly influence the BER performance for channel codes. Polar codes constructed using the ASA approach exhibit this inherent characteristic as well. In ASA, as the codeword length is divided into smaller segments, appropriate allocation of coderate to these individual segments becomes crucial to improve BER performance. Larger codeword lengths can accommodate more information bits due to the increased number of reliable channels induced by channel polarization. Consequently, the distribution of information bits among segments plays a vital role in determining BER performance. Evaluating the coderate for the underlying segments is necessary to improve BER performance to the maximum possible extent. Two RATs are proposed and investigated in the following subsections, namely, equal rate assignment (E-RA) and unequal rate assignment (UE-RA). The Bhattacharya parameter Z=0.5 is used for channel polarization.

#### 3.3.1. Equal Rate Assignment

In E-RA, it is aimed to assign each of the *m* segments the same coderate, which is the same as the overall system coderate $R_{d}$, i.e., the effective coderate $R_{d,t}$, of each segment *t* is the same:

(7) $$R_{d,1}=R_{d,2}=...=R_{d,m}=R_{d}$$

while allocating the same coderate to each segment for E-RA is relatively straightforward, there are cases where the loss of information bits (maximum one bit) might occur to offset the required coderate, hence nominally degrading its BER performance. This arises due to the implementation setup, where the number of information bits *K* may occasionally be a fraction for a given codeword length *N* for given coderate $R_{d}$, since $K=R_{d}\cdot N$. To facilitate implementation, the number of information bits *K* is rounded down to the preceding integer value (floor value) or succeeding integer value (ceiling value). In this document, the floor value for *K* is considered for implementation, thus resulting in the loss of one information bit. To mitigate this effect and maintain the required system coderate, we assign the missing bit to the largest segment, since, it has the highest probability of possessing a high-capacity bit channel, when compared to the smaller segments. This situation is illustrated in the subsequent examples.

In Table 1, an example of E-RA is tabulated. It is observed that for N=200 at $R_{d}=0.5$, the total number of information bits allocated to each of the corresponding segments turns out to be the same as the overall information bits. Thus, none of the information bits are lost during the encoding process.

In Table 2, it is demonstrated that for N=200 at $R_{d}=0.3$, the total number of information bits allocated to segments does not match the required overall information bits. One information bit is lost during encoding due to the consideration of floor values. To prevent this loss, one potential solution is to assign the missing information bit to the largest segment. This is because larger polar codewords are better polarized, which effectively should lead to better error-rate performance (as observed in Figure 1).

#### 3.3.2. Unequal Rate Assignment

UE-RA is another way of rate assignment to segments in ASA. In this scenario, each segment can potentially receive different coderates, given that all segments might have different codeword lengths after segmentation, i.e.,

(8) $$R_{d,1}\neq R_{d,2}\neq ...\neq R_{d,m}\neq R_{d},\;\mathrm{if}\;\;N_{1}\neq N_{2}\neq ...\neq N_{m}$$

The number of information bits assigned to each segment depends on the channel characteristics given as input to the system (in this case Z=0.5). This relationship is crucial for UE-RA, which works on the principle that information bits are transmitted on the bit channels with the highest reliability (or minimum *Z*-values). Therefore, joint selection of reliable bit channels is made among all segments using polarized *Z*-values. Since, the total number of required information bits *K* is calculated as $K=R_{d}\cdot N$, *K*-bit channels with the lowest *Z*-values among all segments are collectively identified to adjust the coderate based on the number of channels selected for transmission of information bits on respective segments, i.e., $R_{d,t}=K_{t}/N_{t}$, where t∈[1,m].

An example of the assignment of information bits for N=200 at $R_{d}=0.3$ is provided in Table 3. Comparing to Table 2, clearly the number of bits assigned to each segment ($K_{t}$) is different for E-RA and UE-RA.

In Table 4, the assigned coderate for each segment is depicted over different system parameter requirements. It has been observed that segments with lower coderates ($R_{d}<0.5$) tend to exhibit higher coderates for larger segments compared to smaller ones. Conversely, at higher overall coderates, smaller segments tend to achieve higher coderates. This phenomenon arises because larger segments undergo significant channel polarization, which concentrates the *Z*-values near the extremes (0 or 1). Consequently, at low coderates, a large portion of the information bits are allocated to highly reliable bit channels within these larger segments, resulting in higher coderates for them. In contrast, at higher coderates, after all reliable bit channels in larger segments are utilized, any remaining information bits are allocated to channels in smaller segments. These smaller segments typically have lower *Z*-values compared to the remaining bit channels in larger segments, leading to higher coderates for the smaller segments. This process of joint selection and allocation continues until all information bits are assigned to their respective bit channel positions.

### 3.4. ASA-Based Polar Decoding

The processing steps of the ASA approach at the receiver side are similar to the steps involved at the transmitter side, i.e., segmentation and aggregation. The only difference is that at the transmitter these steps are used for encoding an input vector from the source, whereas at the receiver it aims at decoding the received channel vector.

**Figure 3:**
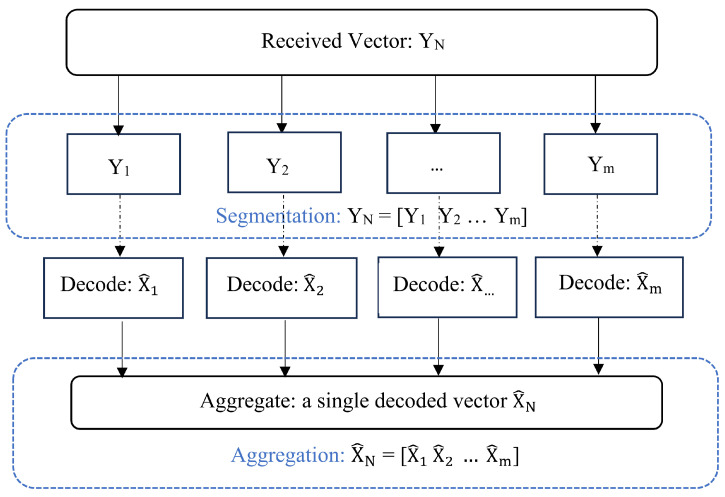
Block diagram for ASA decoding.

Figure 3 shows a block diagram depicting the decoding process performed at the receiver. The received vector $Y_{N}$ is segmented as per the same rules defined for encoding at the transmitter. Since, the segmentation rules are identical for encoder to decoder, the segmentation rules would yield the same parameter values. The resulting segments are then decoded using the SC decoder over the super-generator matrix (5). The decoded vectors ${\hat{X}}_{m}$ of *m* segments are finally aggregated to form the overall decoded output vector. The decoded output vector is then compared with the input vector to evaluate the BER. It is necessary to mention that the arrangement of segments is insignificant. Any arrangement will result in the same BER output, i.e., $\left[N_{1},N_{2}\right]$ is similar to $\left[N_{2},N_{1}\right]$. This is because each segment is processed independently at the transmitter and receiver.

It is assumed that the receiver has prior knowledge about segments:

- Segment lengths $N_{t}$ (where t∈[1,m]);
- Segment coderates $R_{d,t}$ (where t∈[1,m]) along with the RAT.

## 4. Assessment of ASA Technique—Complexity and Error-Rate Performance

In this section, the novel ASA technique proposed in Section 3 is analyzed from two perspectives:

1. Section 4.1 provides detailed analysis on the computational complexity (CC) of ASA compared to downsizing techniques. This is aimed at validating the need and applicability of a new polar code construction technique.
2. Section 4.2 provides comparative BER performance curves against existing code construction techniques (to validate ASA), and thereby over different system parameter values.

### 4.1. Complexity Comparison: ASA vs. Puncturing/Shortening

The computational complexity for decoding is dependent on the total number of mathematical operations performed to decode a given codeword. This section gives a detailed complexity comparison of the proposed ASA technique with that of the downsizing—puncturing and shortening—techniques.

Since we have implemented the SC decoder only, we will use it as a reference to determine the CC, which is stated as follows:

(9) $$CC_{SC}=O\left(N\cdot log_{2}N\right)$$

where *N* is the length of the codeword to be decoded. For puncturing/shortening, if $N_{orig}$ denotes the original codeword length which is punctured/shortened, CC can be simply denoted as

(10) $$CC_{punc/shrt}=N_{orig}\cdot log_{2}N_{orig}$$

The computational complexity of ASA depends on the lengths of its segments. The complexity in ASA is the sum of individual complexities of segments. Therefore, the largest segment contributes the most in overall complexity. For an overall codeword length *N*, which can be segmented into *m* components-$\left\{N_{1},N_{2},\ldots ,N_{m}\right\}$, the overall complexity of the system is expressed as

(11) $$CC_{SC-ASA}=\sum_{t=1}^{m}\left(N_{t}\cdot log_{2}N_{t}\right)$$

The choice of segments for a given codeword length *N* has an impact on the overall complexity. If the choice of segment is made according to the aforementioned segmentation rules, the total number of segments will be less, indicating the presence of largest possible segments. This, however, would increase CC, although the overall complexity would still be much less than the available downsizing schemes. On the other hand, if smaller segments are chosen, the CC will be only slightly lower compared to the recommended design.

**Figure 4:**
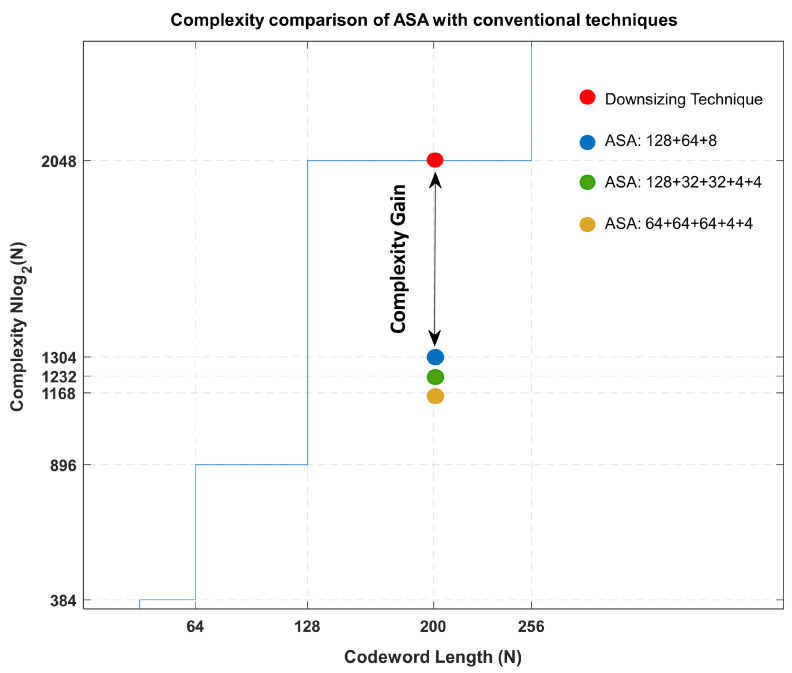
CC comparison of ASA with puncturing/shortening.

Assuming an example for N=200, the CC for various configurations is illustrated in Figure 4:

1. Polar codeword with codeword length 256 bits, downsized (punctured/shortened) by 56, effectively resulting in N=200 bits.
2. N=128+64+8 has fewer segments with large segment lengths. It results in 744 units difference, i.e., gain in CC (≈36%) for puncturing/shortening.
3. N=128+32+32+4+4 has a greater number of segments, with smaller segment lengths as compared to case 1. It results in 816 units difference, i.e., gain in CC (≈40%) for puncturing/shortening.
4. N=64+64+64+4+4 has a greater number of segments, with smaller segment lengths as compared to case 1. It results in 880 units difference, i.e., gain in CC (≈43%) for puncturing/shortening.

### 4.2. Error-Rate Performance

In order to validate the utility of the ASA technique for polar code construction, it is essential to assess the error-rate performance of ASA generated polar codes to conventional and state-of-the-art polar coding techniques. In this section, we provide a validation, and thereafter, comparative analysis of the error-rate performance of the ASA polar codes. To streamline the results and to maintain coherence, we use the codeword length N=200, exemplified in Section 4. All simulations have been performed over an additive white Gaussian noise channel (AWGNC) using binary phase-shift keying (BPSK) modulation scheme.

In Section 4.2.1, BER curves of ASA against different codeword lengths, different segmentation patterns, and a comparison to downsizing techniques are provided. In Section 4.2.2, BER curves generated by ASA for different coderates with different RATs are provided to determine the optimized design technique against the desired system parameters. As mentioned previously, all BER curves are generated using the SC polar decoder.

#### 4.2.1. Validation and Comparative Performance to Conventional Methods

**Figure 5:**
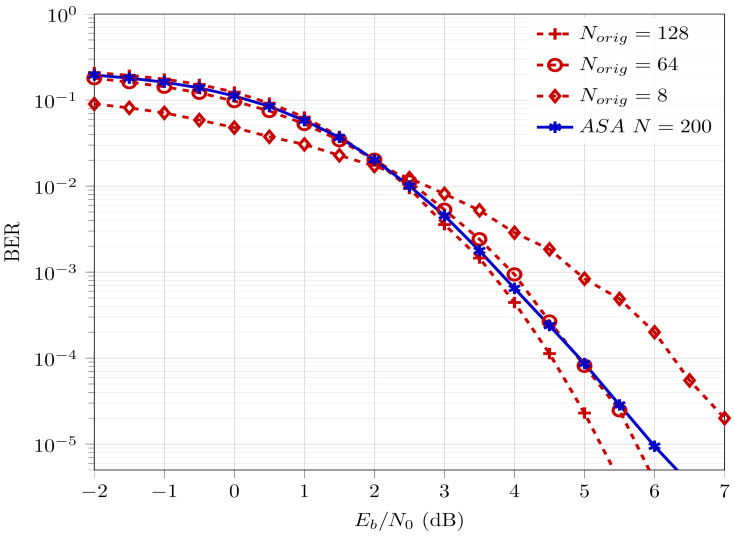
BER comparison for ASA with N=200 against $N_{orig}=128$, $N_{orig}=64$, and $N_{orig}=8$ at $R_{d}=0.5$.

As a first step, N=200 is generated using the segmentation rules, mentioned in Section 3.2, and Figure 2, i.e., with segment lengths $N_{1}=128$, $N_{2}=64$, and $N_{3}=8$ (codeword lengths of the form $N=2^{n}$ generated by 2×2 kernels). The BER of ASA for N=200 with E-RA is compared to the BER of conventional polar codes for codeword lengths $N_{orig}=128$, $N_{orig}=64$, and $N_{orig}=8$. A coderate value of $R_{d}=0.5$ is assumed for all codeword lengths. The BER curves are depicted in Figure 5.

First and foremost, in Figure 5 it is clearly observed that the blue curve has a waterfall behavior, which is a characteristic of the BER for error-correcting channel codes. It achieves a $BER=10^{-5}$ at a bit-energy-to-noise ratio ($E_{b}/N_{0}$) =6 dB. This validates the proposed ASA scheme. The red curves denote BER curves for $N_{orig}=128$, $N_{orig}=64$, and $N_{orig}=8$. Clearly the BER for ASA N=200 is worse than the BER for 128. This indicates that the segment with the longest codeword length would effectively be the performance bound of BER performance for polar codes generated by ASA. In fact, the BER (blue curve) of ASA is the weighted average of BERs of the individual segments (red curves). This is intuitive, since each segment in ASA is encoded/decoded independently, i.e., they do not have any dependence or information exchange whatsoever. This effect can be analytically represented as

(12) $$BER_{ASA}=BER_{Weighted-Average}=\frac{128}{200}\cdot BER_{128}+\frac{64}{200}\cdot BER_{64}+\frac{8}{200}\cdot BER_{8}$$

Thus, the BER curve for the ASA scheme is bounded by the BER curve of the longest and smallest segments, with the largest segment providing the upper bound and the smallest segment providing the lower bound. This suggests that it would be desirable to select the segments with longest codeword lengths to obtain a better BER (although with higher CC, as mentioned in Section 4). This validates the segmentation rules developed in Section 3.2 to achieve the best BER performance.

**Figure 6:**
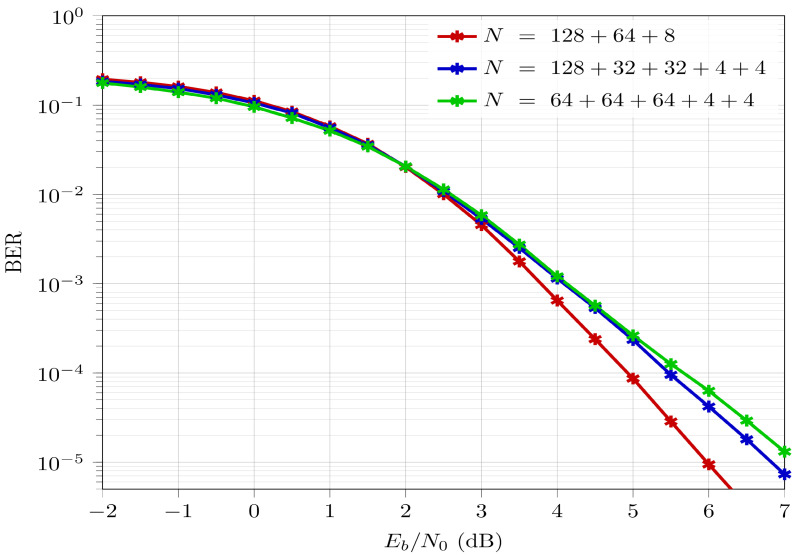
BER comparison for ASA with N=200 at $R_{d}=0.5$ using different segment combinations.

Figure 6 compares the result for N=200 at $R_{d}=0.5$ achieved from different possible choices of segments in the ASA scheme. In this figure, three sets are considered: [128,64,8] (obtained by segmentation rules), [128,32,32,4,4], and [64,64,64,4,4]. These have been implemented to compare the BER performance, also exemplified in Section 4. Evidently, the segments [128,64,8] achieve the best BER performance as compared to alternative selections, outperforming the subsequent one by almost 1 dB. Thus, the presence of a segment with a large codeword length is beneficial for improving BER performance. However, as shown in Section 4, the trade-off is higher CC, specifically by 5.5% for [128,32,32,4,4] segments and 11.6% for [64,64,64,4,4] segments.

**Figure 7:**
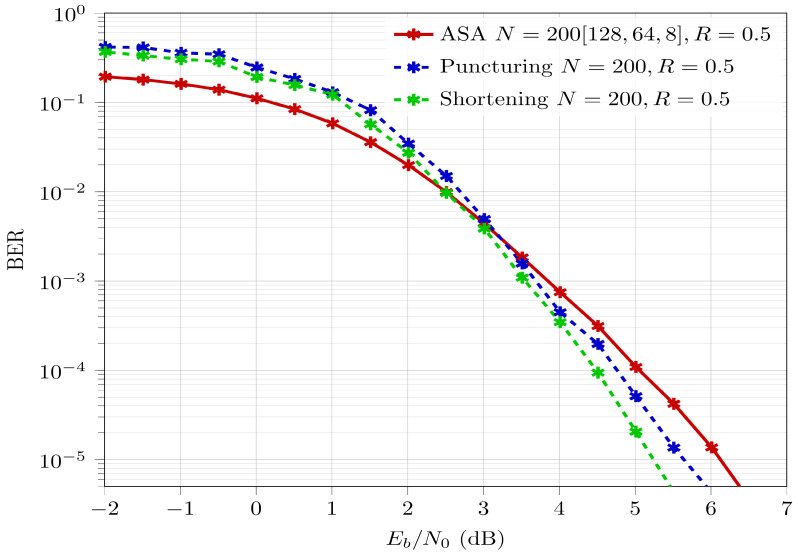
BER comparison for N=200 at $R_{d}=0.5$ generated using ASA against downsized (punctured/shortened) $N_{orig}=256$ codeword.

Figure 7 provides the BER performance of ASA polar codes with N=200 and $R_{d}=0.5$ compared to the BER performance of conventional polar codes with N=200 downsized (punctured and shortened) by 56 bits and $R_{d}=0.5$. The downsizing techniques (puncturing and shortening) used to generate the BER curves are as detailed in [19]. It is observed that the BER for ASA is comparable, i.e., with a BER performance degradation of ≈1.1 dB compared to shortened codes and ≈0.5 dB compared to punctured codes. However, ASA exhibits a reduction in CC by 36%, as mentioned in Section 4. Note that for ASA polar codes, as mentioned in Section 3.2, the BER performance is upper bounded by the codeword length of the longest segment, which in the case of Figure 7 is $N_{orig}=128$. On the other hand the BER performance of downsized polar codes are upper bounded by the codeword length on which downsizing has been performed, which in the case of Figure 7 is $N_{orig}=256$. Based on Figure 1, the capability of ASA for BER performance improvement is clearly less than that of its punctured/shortened counterparts.

#### 4.2.2. Performance of ASA for Different Coderates and Rate Assignment Techniques

**Figure 8:**
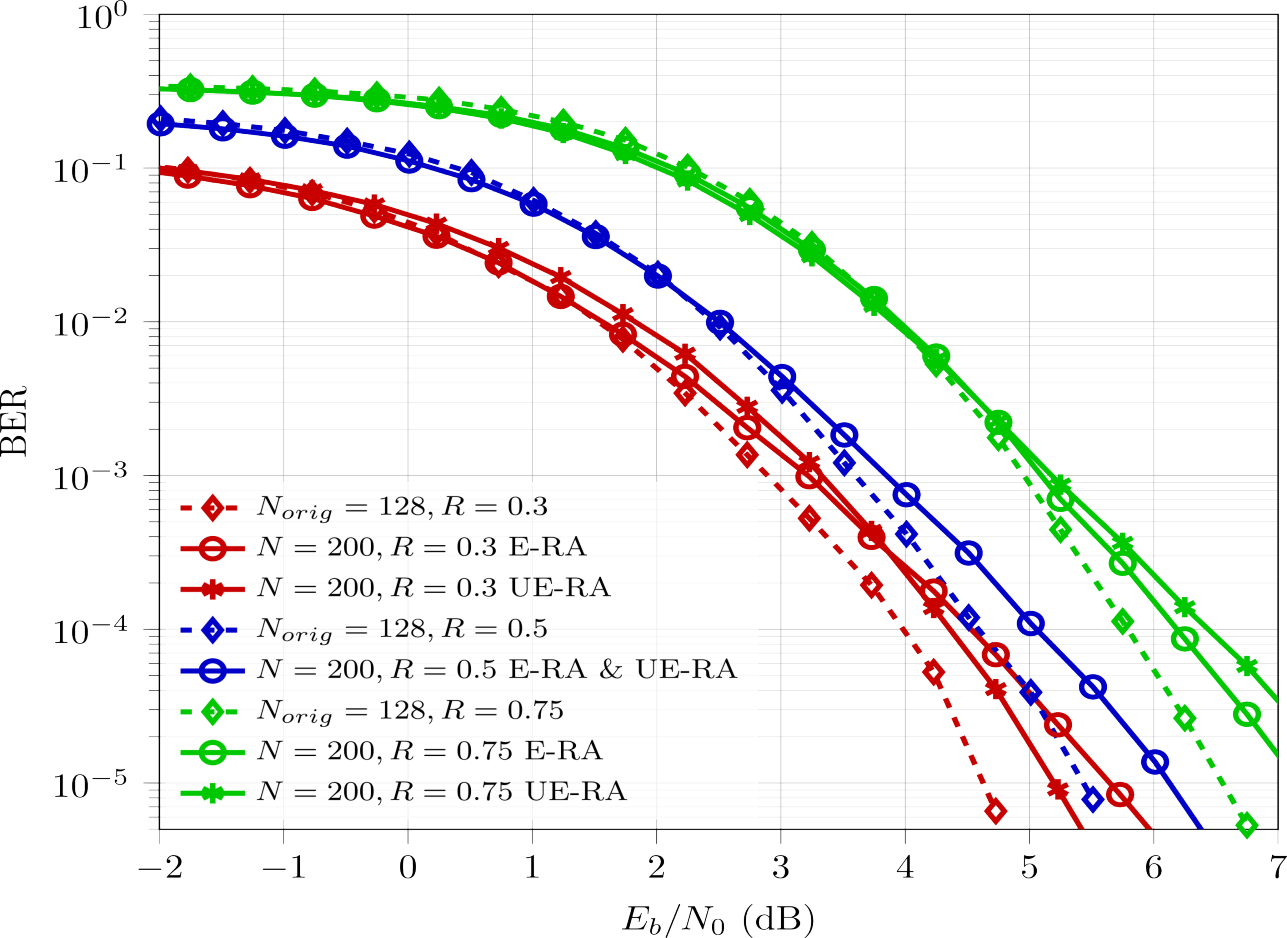
BER comparison of ASA polar codes for N=200 at various coderates with different RATs.

Figure 8 provides detailed error-rate performance comparisons of ASA polar codes for N=200 at various coderates with different RATs (E-RA or UE-RA), while using $N_{orig}=128$ conventional polar codes as a reference. At low coderates, UE-RA provides better BER performance compared to E-RA.

Whereas at high coderates, E-RA outperforms UE-RA because it allows more coderate for bigger segments, which will otherwise, in the case for UE-RA, be allotted to smaller segments, which degrades the overall performance. At half coderate, E-RA and UE-RA do not make any difference because in both cases the selection of bit channels to encode the information bits is exactly the same. Thus, the performance for both RATs at half coderate is identical. From Table 1 and Table 4 one can observe that for $R_{d}=0.5$, E-RA and UE-RA result in the same coderate for each of the segments.

These observations and conclusions affirm the applicability of the proposed ASA scheme and RATs. Consequently, ASA, along with rate adaptation, can be employed to derive any codeword length for the required system parameters, offering reduced complexity and BER performance comparable to downsizing techniques.

## 5. Outlook

ASA has clearly been proven as a competitive polar code construction technique to design variable-length polar codes. Nevertheless, this approach has the potential to be further improved by introducing smarter RATs, that are fine-tuned to additional system parameters or channel conditions. In this paper, we have implemented ASA just for segments generated over 2×2 polarization kernels. This could be easily extended to ASA with segments from higher-order kernel sizes (e.g., 3×3 kernel), MKPC, or non-binary kernels [23]. Additionally, segments can be generated specific to the system requirements, i.e., there is a considerable degree of freedom in the choice of codeword lengths (total and segment-wise, following the segmentation rules from Section 3.2), coderates, underlying polarization kernels, and their combinations. It would be worthwhile to investigate the error-rate performance of ASA over such a wide range of system parameters.

The ASA technique proposed and the corresponding segmentation rules, in this paper, are optimized for BER performance. However, to optimize FER performance capabilities, the ASA design technique needs to be modified with a new set of segmentation rules. This could be investigated as part of a future research work, along with generating and analyzing FER curves for validating the ASA technique.

Due to independent encoding and decoding of the segments in the ASA approach, they can be used for transmission that requires unequal error protection. It would be interesting to implement variations in the aforementioned ideas and analyze the error-rate performances using not just the SC decoder, but also SCL, SCF, BP, and other polar decoding techniques.

Recently, machine learning techniques (e.g., using reinforcement learning) have been applied in polar codes to further reduce their implementation complexity and improve performance. The method proposed in [24] provides a novel technique for polar code construction that no longer depends on sorting and selecting bit channels by reliability. Hence, extending these reinforcement-learning-based algorithms to the ASA scheme would be an interesting research topic.

## 6. Conclusions

The proposed Adaptive Segmented Aggregation scheme can generate polar codes with any arbitrary codeword length with good error-rate performance compared to downsizing techniques, with an overall lower complexity. It is simple to implement and can easily be designed with the existing decoding techniques. Therefore, the ASA technique for polar code construction significantly improves the practical applicability of polar codes owing to the flexibility in integrating a wide range of system parameters. ASA allows for the implementation of any suitable sequence of segments, balancing complexity with BER performance.

This paper proposed a novel construction scheme ASA for polar codes. This technique segments a given codeword of any required codeword length into multiple component codewords that are then encoded or decoded using the conventional approach. In principle, the ASA scheme works for any component segments that can be processed by traditional polar codes; however, in order to obtain optimal error-rate performance, the segmentation rules provided in Section 3.2 must be followed. It has been concluded in Section 4 that the computational complexity of ASA is relatively lower than that of downsizing schemes like puncturing and shortening. This efficiency stems from the need for LLR calculations solely for the desired codeword length in ASA, in contrast to the entire codeword length before punctured/shortened bits are truncated.

The choice of segments clearly has an impact on the BER performance and CC of the ASA approach with respect to puncturing or shortening. Smaller segments have lower CC, but at the cost of lower BER performance. Whereas larger segments have higher CC, but can achieve lower BER performance. This trade-off should be an important consideration when using ASA for polar code construction, since the same codeword can be generated in multiple ways depending on the tolerance of complexity and desired BER.

Rate assignment techniques have been developed for ASA, which determine the allocation of coderates to individual segments. It is observed that at low coderates, larger segments are allocated higher coderates, while smaller segments receive lower coderates. For high coderates, the proposed strategy assigns higher coderates to smaller segments and relatively low coderates to larger segments. These observations suggest using UE-RA at low coderates and until half coderate, while E-RA seems to be the better option for higher coderates. The BER performance of the proposed ASA approach can also be described as the weighted average of BERs of component segments achieved when processed separately using conventional polar codes. In ASA, as segments are processed separately, there is no error propagation among segments, which in turn avoids error aggregation. This makes it suitable for unequal error protection scenarios. These advantages make the novel proposed scheme competitive against downsizing schemes. Overall, it has the potential to extend the practical applicability of polar codes.

## Figures and Tables

**Table 1:** Example of E-RA for N=200 at $R_{d}=0.5$.

Segment Parameters				System Parameters
**Codeword length**	$N_{1}=128$	$N_{2}=64$	$N_{3}=8$	N=200
**Coderate**	$R_{d,1}=0.5$	$R_{d,2}=0.5$	$R_{d,3}=0.5$	$R_{d}=0.5$
**# Info bits**	$K_{1}=64$	$K_{2}=32$	$K_{3}=4$	K=100
$K_{1}+K_{2}+K_{3}=100$ In this case, no information bits are lost as $K=K_{1}+K_{2}+K_{3}$				

**Table 2:** Example of E-RA for N=200, $R_{d}=0.3$.

Segment Parameters				System Parameters
**Codeword length**	$N_{1}=128$	$N_{2}=64$	$N_{3}=8$	N=200
**Coderate**	$R_{d,1}=0.3$	$R_{d,2}=0.3$	$R_{d,3}=0.3$	$R_{d}=0.3$
**# Info bits**	$K_{1}^{\prime }=38.4$	$K_{2}^{\prime }=19.2$	$K_{3}^{\prime }=2.4$	$K^{\prime }=60$
**Floor value of ** $K_{m}^{\prime }$	$K_{1}=38\left(+1\right)$	$K_{2}=19$	$K_{3}=2$	K=60
$K_{1}+K_{2}+K_{3}=60$ In this case, one additional information bit is assigned to the largest segment, i.e., $K_{1}$				

**Table 3:** Example of UE-RA for N=200 at $R_{d}=0.3$.

Segment Parameters				System Parameters
**Codeword length**	$N_{1}=128$	$N_{2}=64$	$N_{3}=8$	N=200
**Coderate**	$R_{d,1}=0.32$	$R_{d,2}=0.2813$	$R_{d,3}=0.125$	$R_{d}=0.3$
**# Info bits**	$K_{1}^{\prime }=40.96$	$K_{2}^{\prime }=18.003$	$K_{3}^{\prime }=1$	$K^{\prime }=60$
**Floor value of ** $K_{m}^{\prime }$	$K_{1}=40\left(+1\right)$	$K_{2}=18$	$K_{3}=1$	K=60
$K_{1}+K_{2}+K_{3}=60$ In this case, 1 information bit is assigned to the largest segment, i.e., $K_{1}$				

**Table 4:** Example of UE-RA for N=200 at different $R_{d}$.

N=200			
System rate ↓	$N_{1}=128$	$N_{2}=64$	$N_{3}=8$
0.3	0.3203	0.2813	0.1250
0.5	0.5	0.5	0.5
0.75	0.7344	0.7656	0.8750

## Data Availability

Data available on request and not publicly accessible due to ownership restrictions.

## Abbreviations

The following abbreviations are used in this manuscript:

AWGNC	Additive white gaussian noise channel
ASA	Adaptive segmented aggregation
BER	Bit error rate
BP	Belief propagation
BPSK	Binary phase-shift keying
CC	Computational complexity
E-RA	Equal rate assignment
FER	Frame error rate
MKPCs	Multi-kernel polar codes
RAT	Rate assignment technique
SISO	Soft-input soft-output
SC	Successive cancellation
SCL	Successive cancellation list
SCF	Successive cancellation flip
UE-RA	Unequal rate assignment
